# NSQIP data collection up to 30 postoperative days is sufficient to capture some complications in orthopedic surgeries

**DOI:** 10.1007/s00590-024-04021-6

**Published:** 2024-06-07

**Authors:** Haleigh M. Hopper, Chase T. Nelson, James R. Satalich, Conor N. O’Neill, Alexander R. Vap

**Affiliations:** 1 Department of Orthopaedic Surgery, Virginia Commonwealth University, 1201 E Marshall St #4-100, Richmond, VA 23298 USA; 2 Department of Orthopaedic Surgery, Duke University, Durham, NC USA

**Keywords:** National Surgical Quality Improvement Program, Complications, Complication timing, Adequate follow-up

## Abstract

**Purpose:**

The primary aim of this study is to determine if the 30-day follow-up period used by the National Surgical Quality Improvement Program (NSIQP) is an appropriate timeframe to capture complications after orthopedic surgeries.

**Methods:**

The 2019 NSQIP data were used. The independent variables were complication type. The dependent variable was days to complication. A Shapiro–Wilk test was used to determine if the data were normally distributed.

**Results:**

271,397 orthopedic cases were included. Myocardial infarction, pneumonia, ventilator over 48 h, progressive renal insufficiency, acute renal failure, stroke, and cardiac arrest had positive skewness and positive kurtosis. Deep incisional surgical site infection (SSI), organ/space SSI, wound disruption, unplanned reoperation one, unplanned reoperation two, readmission two, and readmission three had negative kurtosis and negative skewness. Complications with positive kurtosis and positive skewness are more likely to be confined to the 30-day postoperative period, whereas complications with negative skewness and negative kurtosis may be underreported within the 30-day follow-up.

**Conclusions:**

These findings are useful in their ability to inform future orthopedic research using NSQIP which continues to generate new data for surgeons to consider for their postoperative care and complication management**.**

## Introduction

Retrospective database studies analyzing surgical data are frequently used to increase the evidence for surgical interventions and identify topics for future prospective studies [1–3]. Among these databases, the American College of Surgeons National Surgical Quality Improvement Program (ACS-NSQIP) has been frequently analyzed to comment on complication rates within a 30-day follow-up period [4, 5]. Though orthopedic complications are not confined to a 30-day follow-up period, investigating the day the complications occur as well as the rates of postoperative complications within the 30-day window will provide useful evidence to inform surgeon’s postoperative management strategies [6]. The question of this study is: when do complications arise after orthopedic surgery and is the 30-day follow-up window used by NSQIP sufficient to capture these complications. The secondary aim is to determine if the time to complication in orthopedic surgeries was different from other surgical specialties.

## Methods

### Patient population

The ACS-NSQIP was used to conduct this analysis and was therefore exempt from Institutional Review Board review. NSQIP data were collected by the certified Surgical Clinical Reviewer at each participating site. NSQIP uses a systematic sampling process to determine which cases are included in the database. Patients who underwent surgery from January 2019 to December 2019 were included in this analysis. All patients are followed for 30 postoperative days. NSQIP criteria for case exclusion included minors (patients less than 18 years of age), trauma cases, minor cases, and cases that were returned to the operating room due to a complication from a prior procedure. Cases for this analysis were also excluded if the operative time or length of stay (LOS) was less than or equal to zero. Cases were also excluded if functional status, dyspnea status, sex, or American Society of Anesthesiologists (ASA) class did not have a response. If the principal anesthesia technique was “none,” “not reported,” or “other” the case was excluded.

### Variables

For the primary aim, the independent variable was complication type. The complications examined were superficial incisional surgical site infection (SSI), deep incisional SSI, organ/space SSI, wound disruption, pneumonia, unplanned intubation, pulmonary embolism, ventilator use for greater than 48 h, renal insufficiency, acute renal failure, urinary tract infection, stroke, cardiac arrest requiring cardiopulmonary resuscitation (CPR), myocardial infarction, bleeding transfusion complication, deep vein thrombosis (DVT), sepsis, septic shock, Clostridium difficile (C. Diff) infection, death, readmissions, and unplanned reoperations. For the secondary aim, the independent variable was surgical specialty. Surgical specialty was divided into orthopedic surgery and non-orthopedic surgery. The dependent variable was the time from operation to complication measured in days.

### Statistical plan

SPSS version 28.0.1.1 (Armonk, NY) was used for statistical analyses [7]. Measures of central tendency and distribution were reported for all complications. A Shapiro–Wilk test was used to determine if the data were normally distributed. Independent sample t-tests were used to compare days to complication between orthopedic surgeries and non-orthopedic surgeries. Results were statistically significant if *p* ≤ 0.05.

## Results

A total of 271,397 orthopedic cases (age: 61.44 ± 15.88 years, 45.6% male, BMI: 29.90 ± 8.44 kg/m^2^) were included in the analysis. All complications had non-normally distributed data for days to complication (Appendix 1). This excludes blood transfusion for which a Shapiro–Wilk test could not be performed. The most frequent complication was blood transfusions at 4.28%, while the least frequent complication was acute renal failure at 0.09%. Excluding reoperations and readmissions, the mean days to complication ranged from 1.10 days for blood transfusions to 17.67 days for wound disruption (Table 1). The range for all complications was 0–30 days except for blood transfusions which ranged from zero to four days. Average days to readmission ranged from 14.49 for the first readmission to 22.03 for the third readmission. There was only one case that had a fourth and fifth readmission. Deep incisional SSI, organ/space SSI, unplanned intubation, urinary tract infection, cardiac arrest requiring CPR, and blood transfusions had a mode of zero.

**Table 1:** Days to complication descriptives and test for normality

Complication	*N* (%)	Mean (day)	Median (day)	Mode (day)	Standard deviation	Range	Skewness	Kurtosis	Shapiro–Wilk *p* value
Superficial incisional SSI	2666 (0.98)	15.62	15	14	7.828	0–30	0.113	− 1.009	< 0.001
Deep incisional SSI	471 (0.17)	16.21	17	0	8.250	0–30	− 0.386	− 0.655	< 0.001
Organ/space SSI	1203 (0.44)	13.35	14	0	10.111	0–30	− 0.055	− 1.380	< 0.001
Wound disruption	546 (0.20)	17.67	18	19	8.157	0–30	− 0.400	− 0.714	< 0.001
Pneumonia	1916 (0.71)	7.98	5	3	7.916	0–30	1.205	0.407	< 0.001
Unplanned intubation	649 (0.24)	8.12	4	0	8.746	0–30	1.075	− 0.032	< 0.001
Pulmonary embolism	960 (0.35)	8.95	6	3	8.167	0–30	0.953	− 0.287	< 0.001
On ventilator > 48 h	398 (0.15)	7.52	4	3	7.734	0–30	1.435	1.085	< 0.001
Progressive renal insufficiency	300 (0.11)	8.67	5	3	8.214	0–30	1.057	0.040	< 0.001
Acute renal failure	233 (0.09)	8.58	5	3	8.238	0–30	1.170	0.194	< 0.001
Urinary tract infection	2,633 (0.97)	12.38	11	0	8.644	0–30	0.313	− 1.000	< 0.001
Stroke	406 (0.15)	8.14	4	2	8.247	0–30	1.119	0.098	< 0.001
Cardiac arrest requiring CPR	392 (0.14)	6.70	3	0	8.310	0–30	1.364	0.754	< 0.001
Myocardial infarction	1016 (0.37)	5.84	3	2	7.251	0–30	1.790	2.325	< 0.001
Bleeding transfusions	11,621 (4.28)	1.10	1	0	0.977	0–4	0.360	− 1.001	–
DVT	1372 (0.51)	11.39	10	3	7.870	0–30	0.648	− 0.509	< 0.001
Sepsis	1256 (0.46)	9.38	6	2	9.264	0–30	0.670	− 0.892	< 0.001
Septic shock	454 (0.17)	9.85	7	2	9.219	0–30	0.728	− 0.759	< 0.001
Death	2025 (0.75)	13.02	12	2, 3	8.726	0–30	0.322	− 1.070	< 0.001
Unplanned reoperation 1	4349 (1.60)	15.14	15	21	8.694	0–30	–0.067	− 1.146	< 0.001
Unplanned reoperation 2	400 (0.15)	18.31	20	28	8.096	0–30	− 0.389	− 0.956	< 0.001
Readmission 1	9166 (3.38)	14.49	14	14	8.157	0–30	0.190	− 1.096	< 0.001
Readmission 2	550 (0.20)	20.37	21	28	6.709	1–30	− 0.624	− 0.300	< 0.001
Readmission 3	30 (0.01)	22.03	24	30	7.346	4–30	− 0.904	− 0.083	0.007
C. difficile infection	409 ()	12.27	10	5	8.590	0–30	0.568	− 0.884	< 0.001

**Fig. 1:**
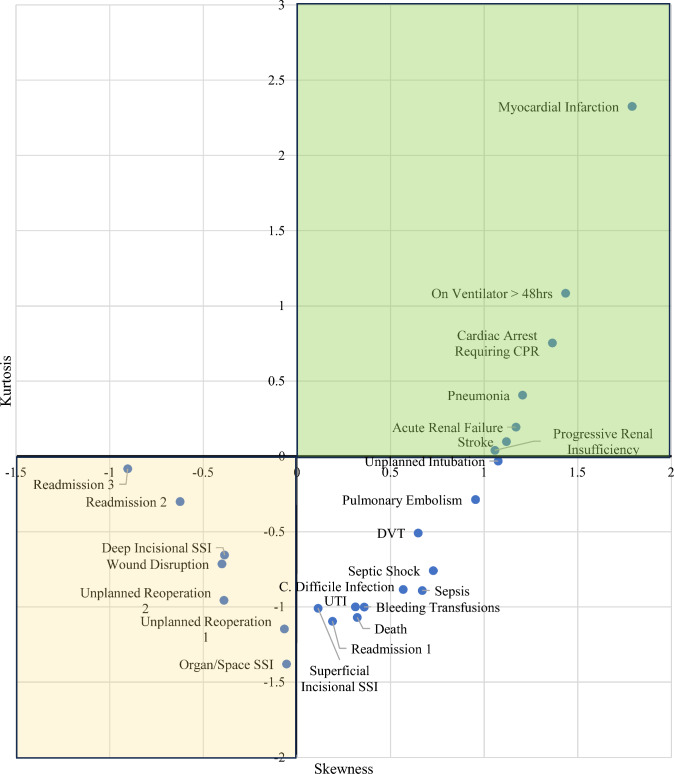
Skewness and kurtosis for each complication (colour figure online)

**Fig. 2:**
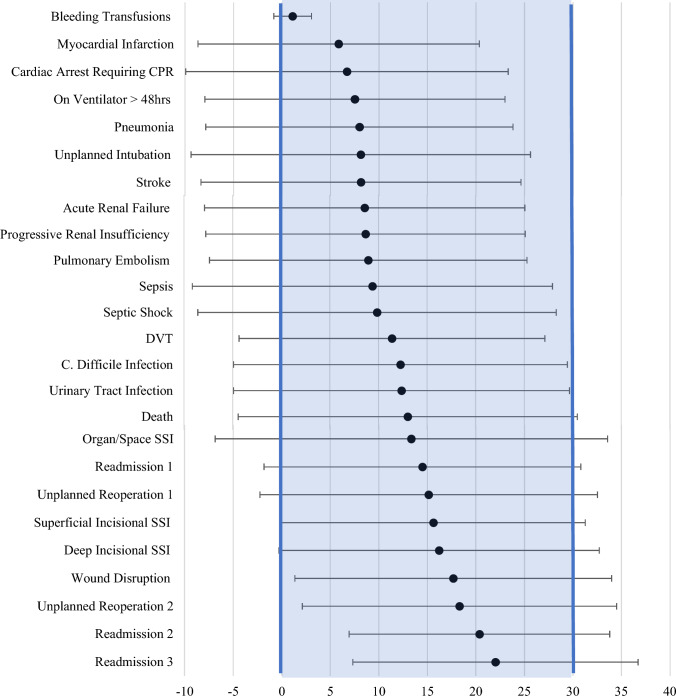
Average day of complication ± two standard deviations

Myocardial infarction, pneumonia, ventilator over 48 h, progressive renal insufficiency, acute renal failure, stroke, and cardiac arrest had positive skewness and positive kurtosis. Complications with positive kurtosis and positive skewness are more likely to be confined to the 30-day postoperative period as shown in the green box in Fig. 1. Deep incisional SSI, organ/space SSI, wound disruption, unplanned reoperation one, unplanned reoperation two, readmission two, and readmission three had negative kurtosis and negative skewness. Complications with a negative skewness and negative kurtosis are less likely to be confined to the 30-day postoperative period as shown in the yellow box in Fig. 1. In addition, complications with an average plus two standard deviations less than 30 days, are more likely to be captured by the NSQIP dataset represented by the blue box in Fig. 2. These complications are blood transfusions, myocardial infarction, cardiac arrest, ventilator over 48 h, pneumonia, unplanned intubation, stroke, acute renal failure, progressive renal insufficiency, pulmonary embolism, sepsis, septic shock, DVT, C. diff infection, and urinary tract infection (UTI).

799,409 non-orthopedic cases (age: 54.79 ± 16.82 years, 41.1% male, BMI: 29.49 ± 8.61 kg/m^2^) were used as a comparison to the orthopedic cases. There was no difference in days to complication between groups for unplanned intubation, progressive renal insufficiency, and cardiac arrest requiring CPR, DVT, readmission two, readmission three, and C. diff infection (Table 2). For complications that reached a statistically significant difference between groups only pulmonary embolism, urinary tract infection, and myocardial infarction were occurring earlier in the orthopedic cases compared to the non-orthopedic cases. For the complications that occurred later in orthopedic cases superficial incisional SSI, deep incisional SSI, organ space SSI, wound disruption, acute renal failure, sepsis, septic shock, death, unplanned reoperation one, and two occurred over one day later (range: 1.54–5.30).

**Table 2:** Days to complication in orthopedic surgeries compared to all other surgeries

Complication	Orthopedic surgery mean ± SD (days)	Non-orthopedic surgery mean ± SD (days)	Difference (days)	*p* value
Superficial incisional SSI	15.62 ± 7.828	14.19 ± 7.408	1.43	< 0.001*
Deep incisional SSI	16.21 ± 8.250	14.05 ± 8.197	2.16	< 0.001*
Organ/space SSI	13.35 ± 10.111	11.24 ± 7.769	2.11	< 0.001*
Wound disruption	17.67 ± 8.157	13.94 ± 7.612	3.73	< 0.001*
Pneumonia	7.98 ± 7.916	7.48 ± 7.041	0.50	0.005*
Unplanned intubation	8.12 ± 8.746	7.59 ± 7.596	0.53	0.10
Pulmonary embolism	8.95 ± 8.167	11.52 ± 8.250	− 2.57	< 0.001*
On ventilator > 48 h	7.52 ± 7.734	4.83 ± 5.469	2.69	< 0.001*
Progressive renal insufficiency	8.67 ± 8.214	9.23 ± 8.460	− 0.56	0.283
Acute renal failure	8.58 ± 8.238	7.04 ± 7.527	1.54	0.003*
Urinary tract infection	12.38 ± 8.644	13.21 ± 8.213	− 0.83	< 0.001*
Stroke	8.14 ± 8.247	7.15 ± 7.664	0.99	0.021*
Cardiac arrest requiring CPR	6.70 ± 8.310	6.95 ± 7.808	− 0.25	0.565
Myocardial infarction	5.84 ± 7.251	6.40 ± 7.529	− 0.56	0.038*
Bleeding transfusions	1.10 ± 0.977	0.66 ± 0.917	0.44	< 0.001*
DVT	11.39 ± 7.870	11.66 ± 7.898	− 0.27	0.289
Sepsis	9.38 ± 9.264	6.14 ± 7.736	3.24	< 0.001*
Septic shock	9.85 ± 9.219	4.55 ± 6.749	5.30	< 0.001*
Death	13.02 ± 8.726	11.21 ± 8.678	1.81	< 0.001*
Unplanned reoperation 1	15.14 ± 8.694	10.33 ± 8.554	4.81	< 0.001*
Unplanned reoperation 2	18.31 ± 8.096	13.99 ± 8.122	4.32	< 0.001*
Readmission 1	14.49 ± 8.157	13.94 ± 7.931	0.55	< 0.001*
Readmission 2	20.37 ± 6.709	20.30 ± 6.797	0.07	0.825
Readmission 3	22.03 ± 7.346	21.50 ± 6.493	0.53	0.679
C. difficile infection	12.27 ± 8.590	11.88 ± 7.922	0.39	0.363

*SSI* surgical site infection, *DVT* deep venous thrombosis

*Indicates statistical significance

## Discussion

The purpose of this study was to discover when surgical complications for orthopedic procedures occur within NSQIP’s 30-day follow-up window as shown in Fig. 2. Recent literature suggests that surgeons may poorly predict their individual complication rates which could be influenced by lack of data regarding when these complications occur [8]. These findings can assist in providing a general gestalt for surgeons to look out for and triage postoperative events in relation to recent surgeries. Though the greater contribution of these findings is to provide context for the increasing body of NSQIP studies that center on orthopedic complications, many of which cite the 30-day follow-up period as a limitation [9]. This study elucidates which of the complications that limitation most truly applies to in NSQIP.

Due to the large sample size analyzed, the use of distribution measures for individual complications allowed for comment on whether the true incidence of these complications is most likely confined within the NSQIP 30-day window. Since each complication distribution is not normally distributed two standard deviations cannot reliably encompass 95% of cases. Therefore, the kurtosis and skewness of each complication can inform the interpretation of the data. Complication distributions with negative kurtosis have a flatter distribution and can be interpreted as occurring with more unpredictable incidence, making a complication less likely to be confined within the 30-day window. Likewise, complications with negative skewness may indicate that the mean incidence is closer to postoperative day 30 than to day zero, thereby reducing confidence of complications being confined to 30 days. Using the same logic, positive skewness and positive kurtosis taken together can increase confidence that such complications are confined within the 30-day follow-up period as depicted in Fig. 1. Using this criteria, myocardial infarction, pneumonia, ventilator over 48 h, progressive renal insufficiency, acute renal failure, stroke, and cardiac arrest’s true incidences are likely confined to 30-day follow-up. Given these findings, the limitation of insufficient follow-up would not apply to these NSQIP complications.

The secondary aim of this study was to determine if the time to complications in orthopedic surgeries was different from other surgical specialties that use NSQIP to investigate complications. Table 2 provides a complete account of these differences, highlighting a greater than 4-day delay in complication onset for unplanned reoperations and septic shock in orthopedic cases. Superficial incisional SSI, deep incisional SSI, organ space SSI, wound disruption, acute renal failure, sepsis, septic shock, death, and unplanned reoperations one and two all occurred over one day later in orthopedic surgeries than in non-orthopedic surgeries. Conversely, pulmonary embolism was the only complication that occurred over one day earlier in orthopedic cases. Though these differences are statistically significant, the clinical significance of these findings remains in question given that many of the differences are a matter of one or two days. Though this appears to be the first study to evaluate general orthopedic surgery complication timelines, studies exist that map out the distribution and timeline of complications for specific procedures [2, 10].

These findings should be weighed in the context of existing NSQIP limitations as a retrospective epidemiological study based on surgical specialty incidence. Most notably, a question of dubious internal validity, with one study recently finding underreported complications related to hip fractures in the database [11]. NSQIP is subject to the selection bias of what data are included, as well as the lack of the context provided by individual patient characteristics and surgical details which would increase the clinical utility of its findings [12]. Without this surgical or clinical context, any profuse discussion on etiologies or potential contributing factors is weakened. Future studies are indicated to identify the inherent differences that give rise to these findings, and whether they are related to different patient or surgical characteristics. The generalizability of this data is confined to cases that fit NSQIP’s inclusion criteria as set forth in the methods section above. Likewise, comment on other orthopedic complications such as nonunion, hardware failure, implant loosening, and others, is limited due to NSQIP not tracking these complications. In addition, the lack of specification of type of orthopedic surgery (upper extremity, lower extremity, or spine) could confound our results.

## Conclusions

This study is an analysis of the NSQIP database to report when complications were occurring within 30-day follow-up for orthopedic surgeries. Interpretation of the analyzed data suggests that myocardial infarction, pneumonia, ventilator over 48 h, progressive renal insufficiency, acute renal failure, stroke, and cardiac arrest can likely be captured by 30-day follow-up. Deep incisional SSI, organ/space SSI, wound disruption, unplanned reoperation one and two, and readmissions two and three may still be underreported by 30-day follow-up. We also identified differences between average times to complication for orthopedic surgeries compared to non-orthopedic surgeries, though further work is required to identify whether these findings have any clinical significance. This study is limited in its ability to comment on the etiology of these differences as it is retrospective and general in its analysis. Any differences are likely multifactorial and provide opportunity for further investigation into etiology of existing differences. These findings are useful in their ability to inform future NSQIP orthopedic research and generate new data from a large sample for surgeons to consider for their postoperative care and complication management.
